# Feasibility and transcriptomic analysis of betalain production by biomembrane surface fermentation of *Penicillium novae*-*zelandiae*

**DOI:** 10.1186/s13568-017-0529-4

**Published:** 2018-01-08

**Authors:** Hailei Wang, Yi Li, Kun Zhang, Yingqun Ma, Ping Li

**Affiliations:** 1 Henan Province Engineering Laboratory for Bioconversion Technology of Functional Microbes, College of Life Sciences, Henan Normal University, Xinxiang, 453007 China; 2 Advanced Environmental Biotechnology Center, Nanyang Environment and Water Research Institute, Nanyang Technological University, Singapore, 637141 Singapore

**Keywords:** Betalains, Biomembrane surface cultivation, *Penicillium novae*-*zelandiae*, Tyrosine, RNA sequencing

## Abstract

In this study, a biomembrane surface fermentation was used to produce red pigments of *Penicillium novae*-*zelandiae*, and the significant improvement in pigment production by the addition of 0.4 g/L of tyrosine demonstrated that the red pigments probably contained betalain. Therefore, one red pigment was purified, and identified as 2-decarboxybetanin by high-resolution mass spectrometry (MS) and MS/MS analysis. Transcriptomic analysis revealed the differentially expressed genes and metabolic profile of *P. novae*-*zelandiae* in response to different cultivations and exhibited the complete biosynthetic pathway of 2-decarboxybetanin in *P. novae*-*zelandiae*. Betalains are important water-soluble nitrogen-containing food coloring agents, obtained mainly from beetroot by chemical extraction. This paper is the first report about the production of betalain by microbial fermentation, and results exhibit the possible use of fungal fermentation in future 2-decarboxybetanin production.

**Electronic supplementary material:**

The online version of this article (10.1186/s13568-017-0529-4) contains supplementary material, which is available to authorized users.

## Introduction

Pigments, including synthetic and natural pigments, are widely used in food, brewing, and cosmetic industries. Synthetic pigments were extensively used in the past because of their bright color, high stability, and low cost. However, most of them are harmful to human health in varying degrees, and many people interpret the content of chemical pigments as a contaminant. Therefore, synthetic pigments have been prohibited or strictly limited in many countries. Natural pigments hold advantages over synthetic pigments in terms of nutritious and pharmacological functions (Arad and Yaron 1992). With the continuous improvements in the quality of life, the demand for natural pigments is growing rapidly. In Japan, China, and the United States, the numbers of natural pigments allowed are 97, 48, and 30, respectively (Mapari et al. 2005, 2010).

Betalains are a group of water-soluble plant pigments that are used as food colorants. They are ammonium derivatives of betalamic acid and present in considerably high amounts in certain foodstuffs, including red beets, prickly pear fruits, and Amaranthus seeds (Strack et al. 2003). These pigments have no toxic effects on the human body; as important commercial color additives, no upper limit is necessary for the recommended daily intake of betalains (Delgado-Vargas et al. 2000). More interestingly, very few pharmacological applications of betalains exist. Recently, they have received attention because they show antiviral, antioxidant, and antimicrobial activities (Manohar et al. 2017; Sreekanth et al. 2007; Kanner et al. 2001). Beet roots currently represent the main commercial source of betalains. Thus, the high pigment content in beetroots is crucial. Recent efforts are centered around the betalain content in red beets through selective breeding, because the average pigment content in beets is approximately 130 mg/100 g fresh weight (Delgado-Vargas et al. 2000; Sekiguchi et al. 2013). Betalains can be extracted from plant roots with pure water, at room (or reduced) temperature. But the use of methanol or ethanol solutions in most cases is necessary to achieve complete extraction. Chemical extraction is characterized by high production cost and serious environmental pollution. Agricultural fields used for planting beets are diminishing with the decrease in arable land, and the cost associated with overcoming of environmental pollution is unsustainable for manufacturing enterprises because betalains are inexpensive products. Therefore, the search for a novel production method with low cost and high productivity has become an important issue for betalain production (Pavlov et al. 2005; Moreno et al. 2008).

In this study, biomembrane surface fermentation was used to produce the potential red pigments of *Penicillium novae*-*zelandiae* (Wang et al. 2011). A betalain, 2-decarboxybetanin, was separated from the pigments and then identified by high-resolution mass spectrometry. This work aimed to (i) investigate the feasibility of the production of betalain by fungal fermentation and (ii) reveal the biosynthetic pathway of 2-decarboxybetanin in *P. novae*-*zelandiae* by RNA sequencing.

## Materials and methods

### Microorganism and chemicals

*Penicillium novae*-*zelandiae* HSD07B (CCTCCM2012198) was obtained from the Henan Province Engineering Laboratory for Bioconversion Technology of Functional Microbes, Henan Normal University, Xinxiang, China. The fungus was stored on potato dextrose agar (PDA) plate at − 20 °C before use. All the chemicals used were of spectral or analytical grade unless otherwise stated.

### Shake-flask cultivation and biomembrane surface cultivation

*Penicillium novae*-*zelandiae* was grown on potato dextrose agar for 3 days at 28 °C before harvesting the spores using a camel hairbrush, and spore suspension was prepared in sterile water. Shake cultivation was performed according to the following procedure: four 500 mL Erlenmeyer flasks each containing 200 mL of modified Czapek Dox liquid medium (10.0 g/L glucose, 3.0 g/L NaNO_3_, 1.0 g/L K_2_HPO_4_, 0.5 g/L MgSO_4_, 0.5 g/L KCl, and 0.01 g/L FeSO_4_) were inoculated with a 0.5 mL aliquot of spore suspension (3.7 × 10^7^ spores/mL). The flasks were then incubated in a thermostat shaker at 150 rpm and 28 °C. The procedure for biomembrane surface cultivation was the same as that of shake cultivation except that the rotary speed of the thermostat shaker was at 50 rpm. Color value (CV) of pigment solution was defined as the optical density value and determined at the maximum absorption wavelength of water solution of the red pigment, namely, 505 nm. In addition, the fermentation broth was filtered using filter paper (Grade 1:11 lm, Whatman, UK), the cell-free filtrate was mixed with ethanol (filtrate:ethanol = 1:1.5) and the mixture was subjected to centrifugation at 2600×*g* for 10 min. The supernatant was dried in a rotary evaporator at 50 °C and the crude pigment was mixed with 100 mL petroleum to remove hydrophobic substances. The remaining red pigment was dried as standard to create the calibration curve. The concentration of red pigment was calculated according to the regression of Eq. (1) (Wang et al. 2011):

1 $$Y = 1.4491X + 0.0130\quad (R^{2} = 0.9998),$$

where *Y* is the concentration of red pigment (g/L), and *X* is the OD of the pigment solution at 505 nm.

### Effect of amino acids on red pigment production

The effects of 12 amino acids, namely, Glutamic acid (Glu), Aspartic acid (Asp), Arginine (Arg), Proline (Pro), Valine (Val), Isoleucine (Ile), Glycine (Gly), Alanine (Ala), Serine (Ser), Lysine (Lys), Histidine (His), and Tyrosine (Tyr), on pigment production were evaluated. Amino acids at a dosage of 0.5 g/L were added to the modified Czapek Dox liquid medium, and the fermentation process was the same as that of biomembrane surface cultivation described previously. Another Tyr dosage test was also conducted. In this test, we added 0, 0.2, 0.4, 0.6, 0.8, and 1.0 g/L of Tyr to 500 mL flasks containing 200 mL of modified Czapek Dox liquid medium. Biomembrane surface cultivation was conducted at 28 °C in a thermostat shaker. During the tests, CV and the concentration of red pigment were measured daily.

### Identification of pigment component

The fermentation broth was filtered using filter paper (Grade 1:11 µm, Whatman, UK). The cell-free filtrate was mixed with ethanol (filtrate:ethanol = 1:1.5), and the mixture was centrifuged at 2600*g* for 10 min. The supernatant was dried in a rotary evaporator at 50 °C, and the crude pigment was mixed with 100 mL of petroleum to remove hydrophobic substances. The remaining red pigment was used as a sample for silica gel (200 mesh) column chromatography eluted with 1-butanol: ethanol: petroleum ether (2:2:6, V/V). A collected component of red pigment was analyzed by silica gel thin-layer chromatography (TLC) using 1-butanol: ethanol: water (3:5:2) as mobile phase. A high-resolution electrospray mass spectrometer (MicrOTOF-Q II, Bruker Daltonics Corporation, USA) was operated in negative ion mode for mass spectrometer analysis. The capillary voltage was set at 4500 V with an end plate offset potential of 500 V. Data were collected from 50 to 1000 *m/z* with an acquisition rate of 1 spectrum per second. The dry gas was set to 1.2 L/min at 120 °C with a nebulization gas pressure of 0.4 bar. In addition, HPLC-MS/MS analysis was conducted on an Esquire plus 3000 (Bruker Daltonics, Billerica, MA, USA) ion trap mass spectrometer with an electrospray interface (ESI) utilizing HPLC eluted with the mobile phase consisted of 25 mM NH_4_OAc as well as 25 mM NH_4_OH in water, and acetonitrile (90:10). The analysis was conducted in positive-ion mode and operated according to defined conditions: nitrogen gas temperature, 320 °C; drying gas flow rate, 7 L/min; capillary voltage, 4500 V; nebulizing pressure, 27 psi. Mass spectra were recorded using the full scan mode in the range of 200–800 Daltons.

### Sample preparation, RNA extraction, and RNA sequencing

All samples, including the cultures from biomembrane surface cultivation at hour 36 (T1) and hour 96 (T2) and from shake cultivation at hour 36 (Ck1) and hour 96 (Ck2), were prepared. These samples were immediately frozen in liquid nitrogen and then stored at − 80 °C until RNA isolation. Total RNA was extracted using a Trizol reagent according to the manufacturer’s protocol (Invitrogen, China) and then treated with DNase to remove DNA contamination. The yield and purity of RNA sample were checked using a NanoDrop™ 2000 spectrophotometer (Thermo Scientific, USA) at 260 and 280 nm. The integrity of all RNA samples was assessed by 1.0% agarose gel. The mRNA from total RNA was isolated and enriched using oligo (dT) magnetic beads (Illumina, CA, USA). Subsequently, mRNA was fragmented to short fragments to be used as templates for random hexamer-primed synthesis of first-strand cDNA by fragmentation buffer. Second-strand cDNA was synthesized using buffer, dNTPs, RNase H, and DNA polymerase I. A paired-end cDNA library was synthesized using a Genomic Sample Preparation Kit (Illumina, CA, USA) according to the manufacturer’s instructions. Short fragments were purified with a QIAQuick1 polymerase chain reaction (PCR) extraction kit (Qiagen, Germany) and eluted in 10 µL of elution buffer. An Agilent 2100 Bioanalyzer (Agilent Technologies, Santa Clara, CA, USA) and ABI Step One Plus Real-Time PCR System (Applied Biosystems, Foster City, CA, USA) were used to examine the quality and quantify the sample library (Firon et al. 2013). Finally, cDNA libraries were sequenced on an Illumina HiSeq™ 2500 (Novogene, Beijing, China).

### Gene annotation and normalized expression levels

Raw reads were cleaned by removing adapter sequences, empty reads, and low quality sequences. For annotation analysis, unigenes were BLASTX-searched against five databases, namely, the National Center for Biotechnology Information (NCBI) nonredundant (NR) protein sequence database, the NCBI NR nucleotide sequence (NT) database, Kyoto Encyclopedia of Genes and Genomes (KEGG) orthology database, Swissprot, and PFAM database, using a cut-off E-value of 10^−5^. To eliminate the influence of different gene lengths and sequence discrepancies on expression calculations, gene expression levels based on read counts obtained by RSEM (version v1.2.15) were normalized using FPKM (fragments per kilo bases per million fragments) transformation (Li and Dewey 2011). The calculated gene expression levels were used for direct comparison among samples. Expression values were standardized across the dataset to enable the data from different genes to be combined.

### Screening of differentially expressed genes (DEGs) and KEGG analysis

Using the R package DEGseq, DEGs were identified with a random sampling model on the basis of the read count for each gene at different developmental stages. False discovery rate ≤ 0.05 and absolute value of |log2Ratio| ≥ 1 were set as the threshold for significance of gene expression differences between adjacent samples. The KEGG database was used to assign and predict putative functions and pathways associated with the assembled sequences (Baba et al. 2012; Xie et al. 2011).

### Quantitative real-time PCR

To validate RNA-seq differential gene expression data, six genes related to betalain biosynthesis (DDC, TYR, and COMT), pentose phosphate pathway (PFK), tyrosine biosynthesis (GOT1), and glycolysis (galM) were randomly chosen for validation using real-time quantitative PCR (RT-qPCR). The primers that were designed with Primer software (version 5.0) based on the assembled transcriptome were used for amplification, and the annotations of the products are listed in Table 1. β-Tubulin was used as an internal reference gene, and relative gene expression levels were calculated using the comparative Ct method (Livak and Schmittgen 2001). RT-qPCR analyses were run in triplicate with three biological replicates.

**Table 1:** Primers of the selected genes and internal reference gene

Genes	Forward primer	Reverse primer
*DDC*	AGCCCTCGTCATTCCCGTCTT	TCGCCAACTCCGTCCTCGTAT
*COMT*	CCACCCGTAAACCATCCACAG	CAATCCAGCGAGGTCAATCAA
*TYR*	ATCCGGTGGAGTCAACTACGA	ACTGAGCAAGTGCCTGAATGT
*PFK*	AATGATTACAGCGGCAACTCG	TCGCCAACTCCGTCCTCGTAT
*GOT1*	TCTGAAACAGGCACGGTAATC	TGAACTTTCTCGGCAAATAGG
*galM*	GCTGACCACACCTAGCTTTGG	AGGGAACCTTAGGCAGCATGT
β-tubulin	GTTCTGGACGTTGCGCATCTG	TGATGGCCGCTTCTGACTTCC

### Data availability and statistical analysis

The data sequenced in this study have been submitted into the NCBI Sequence Read Archive under the accession number of SRP114884. All experimental data in this work were presented as the mean ± standard error of the mean and evaluated using one-way ANOVA followed by the least significant difference test, with P < 0.01 and P < 0.05 (SPSS 16.0 for Windows).

## Results

### Biomembrane surface cultivation and shake cultivation

**Fig. 1:**
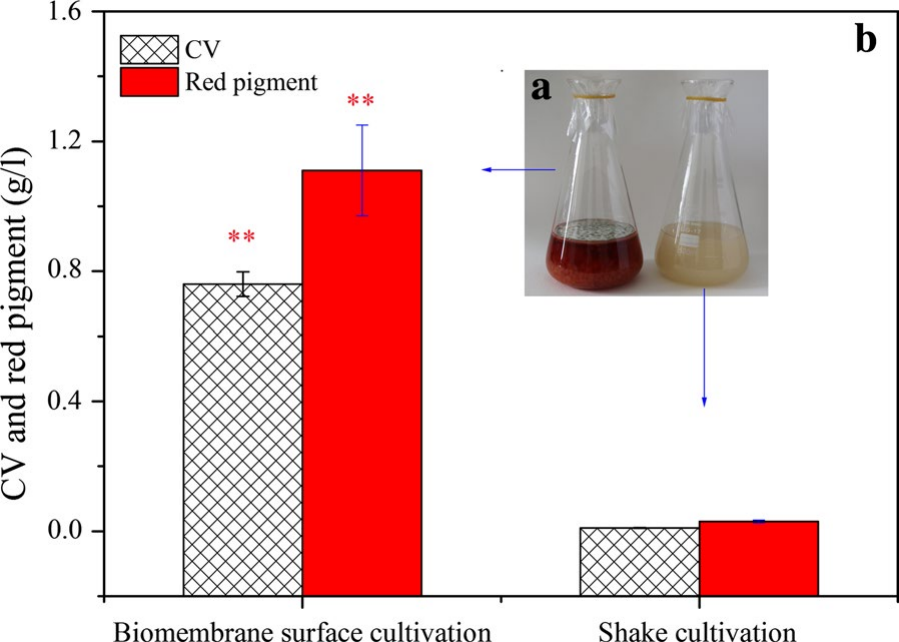
Shake cultivation and biomembrane surface cultivation for production of red pigments of *P. novae*-*zelandiae*. **a** Color comparison and **b** the maximum color value (CV) and concentration of red pigment on day 8

The red pigment produced by *P. novae*-*zelandiae* was proven to be a safe red pigment potentially useful for coloring applications (Wang et al. 2011, 2012). In this work, both biomembrane surface cultivation and shake cultivation were used to produce the pigment. Shake cultivation was not suitable for pigment production because no red pigment appeared in the fermentation broth (Fig. 1). During biomembrane surface cultivation, the CV and concentration of red pigment on day 8 reached 0.76 and 1.16 g/L, respectively, which were significantly higher than those of shake cultivation (*P* < 0.01). However, biomembrane surface cultivation also had an obvious limitation. The formation of biomembrane was a basic precondition for pigment production. Approximately 4 days were required for biomembrane formation, which led to a long fermentation period (8 days) and a low productivity of 0.15 g/L/day.

### Effect of amino acids on pigment production

**Fig. 2:**
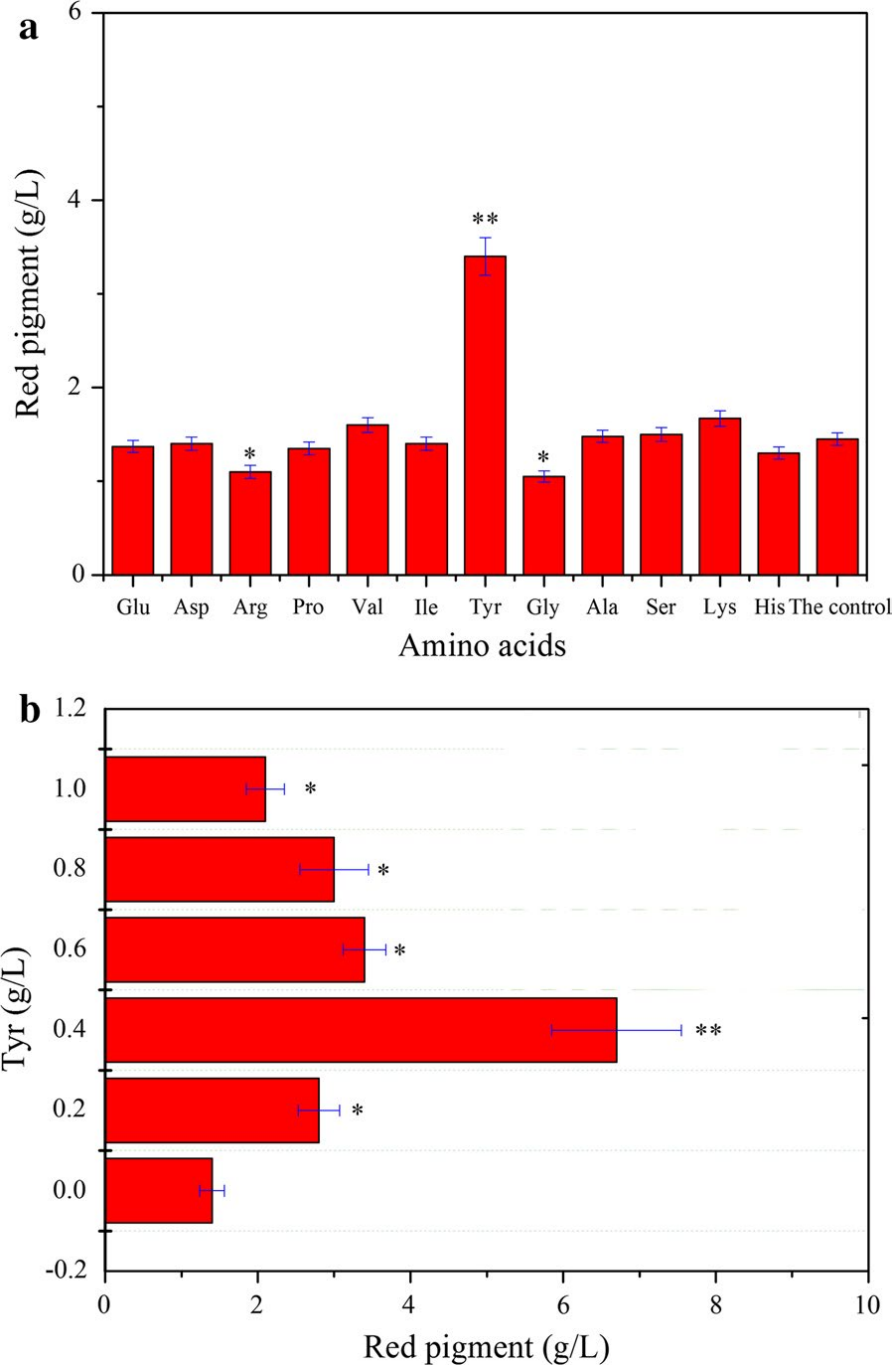
Effect of amino acids on the pigment production of *P. novae*-*zelandiae*. **a** Effect of different amino acids on red pigment production; and **b** the pigment yield varied with Tyr dosage

Amino acids influence natural pigment production considering that they are always involved in the synthetic pathways of natural pigments and function as the precursor of these pigments (Stintzing and Carle 2004; Jain and Gould 2015). The effects of 12 amino acids on pigment production were investigated. Except Tyr, none of these amino acids could improve the pigment yield during biomembrane surface cultivation, and both Arg and Gly resulted in a decrease in pigment content compared with the control (Fig. 2). Interestingly, Tyr significantly improved pigment production (P < 0.01). Further optimization showed that a concentration higher than 0.6 g/L resulted in the decrease in pigment yield, and 0.4 g/L Tyr was an ideal dosage for pigment production. The maximum concentration of red pigment, 6.70 g/L, was 5.78 times higher than that in the initial fermentation. This result indicates that the red pigments composed of at least five red components (Wang et al. 2011) probably contained betalain(s).

### Identification of a red pigment component

**Fig. 3:**
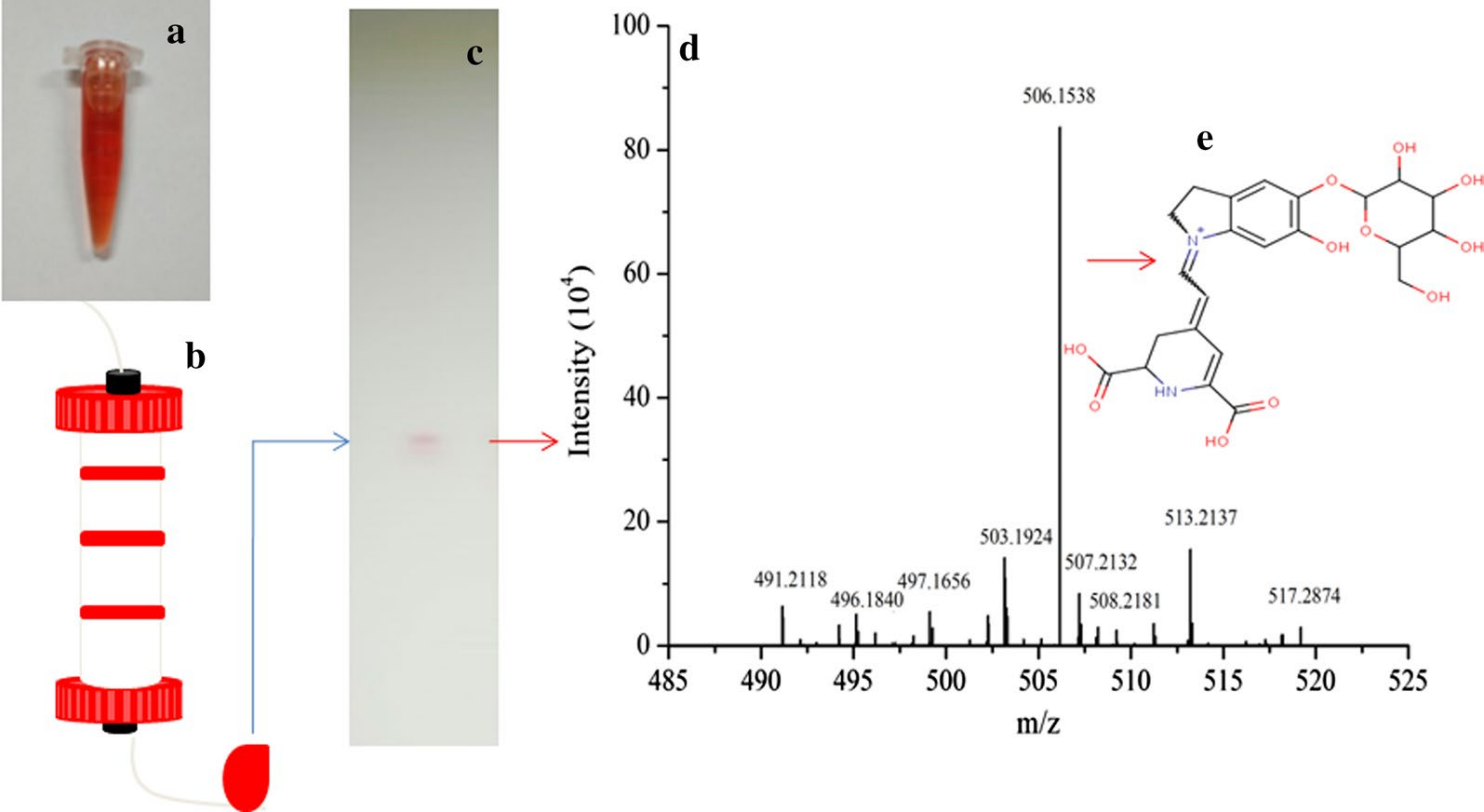
The preliminary separation and identification of 2-decarboxybetanin. **a** The pigment solution before separation; **b** silica gel column chromatography; **c** the pigment component analyzed by TLC; **d** the pigment component identified by high-resolution MS; and **e** chemical structure of 2-decarboxybetanin

Mass spectrometry is the fastest and most effective profiling method to screen and identify natural products, and high-resolution mass spectrometry is extensively used to identify known pigments (Mapari et al. 2005, 2009; Zhang et al. 2012). In this work, a component of red pigment was separated preliminarily using silica gel column chromatography (Fig. 3a, b). TLC analysis revealed that the component mainly contained a red compound (Fig. 3c). The molecular ion of the component [M−H]^−^ well matched the mass spectrum of 2-decarboxybetanin (Delta = 1 ppm) in HMDB (http://www.hmdb.ca/). In negative-ion mode (Fig. 3d, e), the calculated *m/z* from the molecular composition of 2-decarboxybetanin was 506.1542, and 506.1538 Da was found (Lin et al. 2010). In addition, data from HPLC- ESI- MS/MS analysis also support the result of high-resolution mass spectrometry, and both a protonated molecular ion [M + H]^+^ at *m/z* 508.45 and the subsequent fragmentation ion at *m/z* 345.29 (loss of glucose moiety) were found, confirming the presence of 2-decarboxybetanin in fermentation broth of *P. novae*-*zelandiae* (Additional file 1: Table S1).

### RNA sequencing datasets

Table 2 shows a summary of RNA-Seq data quantity. After the quality check, the sequencing of four cDNA samples of *P. novae*-*zelandiae* correspondingly yielded 40,102,094–58,194,514 clean sequences. Good quality scores of the sequences were calculated, and the Q20 and Q30 percentages were higher than 97.83 and 89.08%, respectively (Zhang et al. 2017). In addition, the 0.03% of error rates of sequencing showed that the sequencing results were sufficient and reliable.

**Table 2:** Summary of RNA sequencing clean data

Samples	Sequences	Bases (bp)	Error (%)	Q20 (%)	Q30 (%)	GC (%)
Ck1	53392612	4959651357	0.0302	97.98	89.66	49.95
Ck2	46192964	4264538458	0.0308	97.87	89.22	49.85
T1	58194514	5378836700	0.0303	97.94	89.56	50.17
T2	40102094	3671110092	0.0312	97.83	89.08	50.47

### Analysis of DEGs

**Fig. 4:**
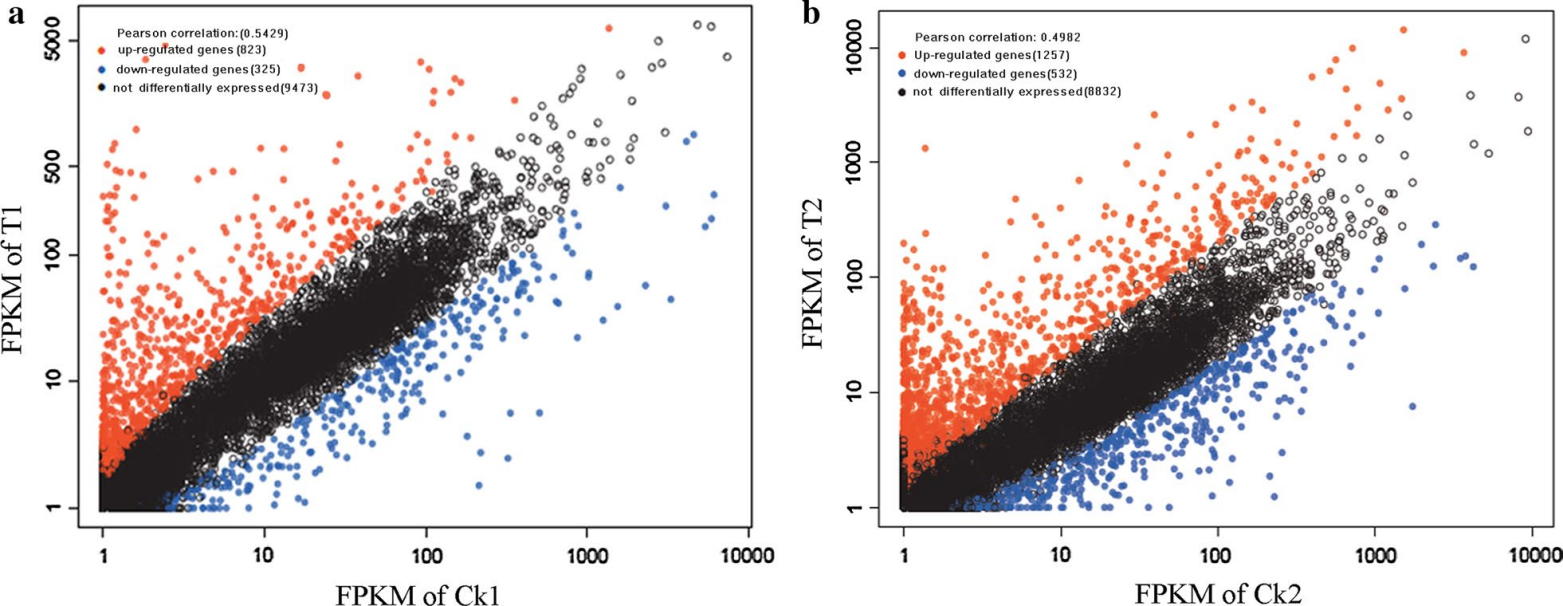
The scatter-plots of differentially expressed genes from transcriptomes of samples. **a** Ck1vs T1, and **b** Ck2 vsT2

To understand better the variety of genes in *P. novae*-*zelandiae* under different cultivation conditions, the DEGs in different samples were determined and visualized by calculating the FPKM value of genes (Li and Dewey 2011). The scatter plots in Fig. 4a, b show that the Pearson correlation coefficients of samples Ck1 versus T1 and Ck2 versus T2 were 0.5429 and 0.4982, respectively. These findings suggest that differences in gene expression between the corresponding samples were significant. At hour 36, the up-regulated and down-regulated genes in T1 versus Ck1 were 823 and 325, respectively; at hour 96, both of them (up-regulated genes, 1257, down-regulated genes, 532) increased. These values show that different cultivation strategies caused significant changes in gene expression.

## Discussion

### Feasibility analysis

2-decarboxybetanin is a derivative of betanin, which is the main component of commercial betalains–beetroot pigments. This study is the first report about the production of betalain by the *P. novae*-*zelandiae* fermentation, although betalains also appear in the fruiting bodies of some higher fungi, including *Amanita*, *Hygrocybe*, and *Hygrosporus* (Strack et al. 1993). Using the 2-decarboxybetanin we purified as the standard substance, the yield of 2-decarboxybetanin produced by *P. novae*-*zelandiae* was evaluated. The result shows that the pigment content is 1.5 g/L, which accounts for approximately a quarter of total red pigments. The average pigment content of beets is approximately 1.3 g/kg fresh weight. Therefore, betalain productivity by microbial fermentation is higher than that by conventional method of extraction from beets because microbial fermentation is easier for industrial-scale production than beets planting. Thus, microbial fermentation for the pigment production has advantages over conventional chemical extraction in production cost and productivity (Stahmann et al. 2000). In addition, in consideration of the decrease in arable land, and cost of environmental pollution worldwide, it is feasible and potential to produce betalain by the microbial fermentation characterized by low cost and high productivity.

### KEGG pathway analysis

**Fig. 5:**
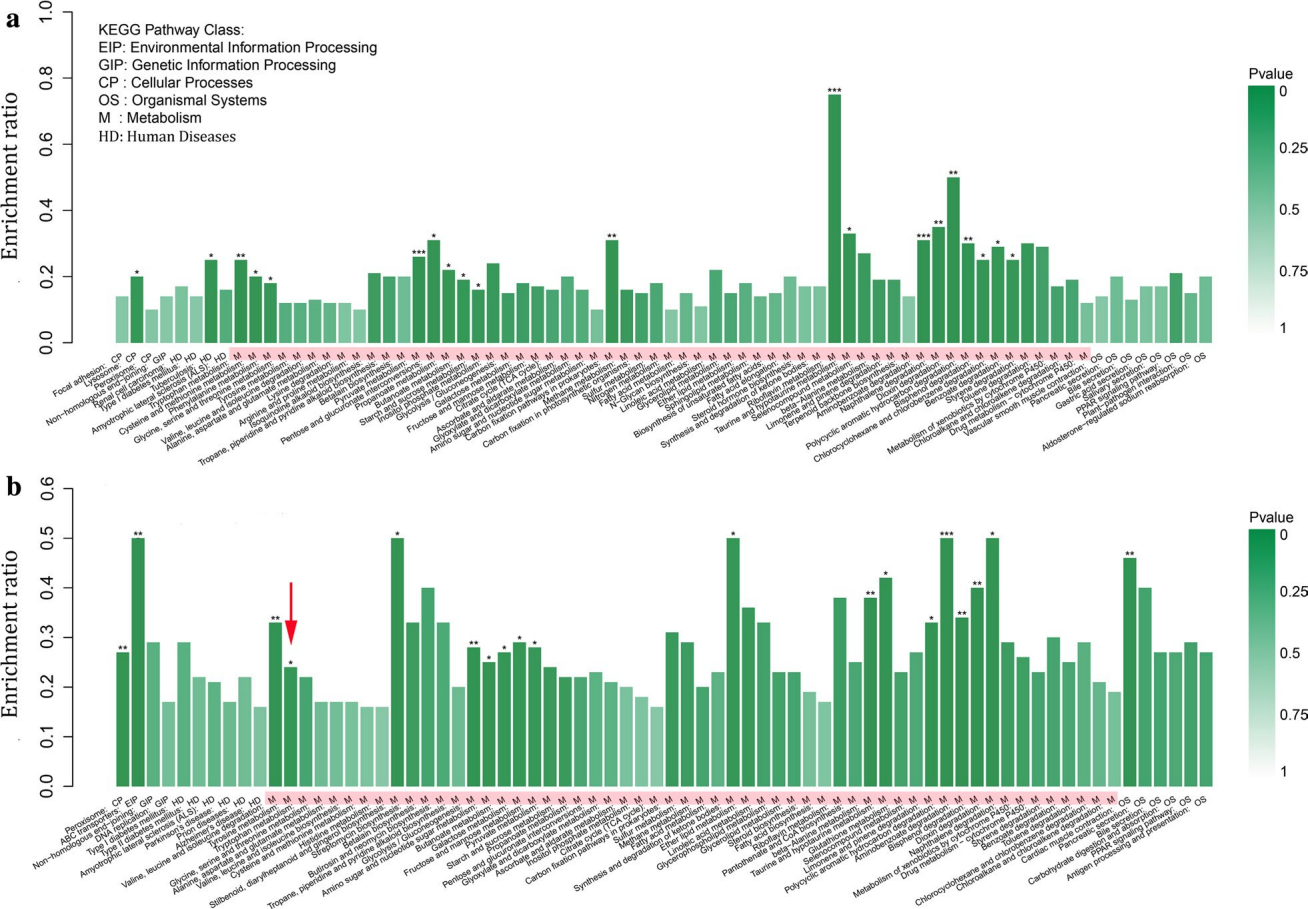
KEGG pathway enrichment analysis of DEGs. **a** DEGs in T1 vs Ck1, and **b** DEGs in T2 vs Ck2

KEGG is the major public database used for pathway analysis. Pathway-based analysis does not only elucidate the biological functions of genes but also further identifies significantly enriched metabolic pathways or signal transduction pathways in DEGs against the whole genome background (Zhang et al. 2012; Wymelenberg et al. 2010). After KEGG pathway analysis, the DEGs were generally placed into six main categories: environmental information and processing, human diseases, cellular processes, organismal systems, genetic information processing, and metabolism. Figure 5a, b show that more DEGs were enriched into the metabolism category than into the other categories. This result suggests that the most significant change occurred in the fungal metabolic profile, although other multiple biological processes were also involved in response to cultivations. At hour 96, enrichment ratios of DEGs, involved in many amino acids metabolism including tyrosine metabolism (red arrow in Fig. 5b) in T2, were significant in comparison with those in Ck2, and these metabolism changes explain why the red pigments only appeared during biomembrane surface cultivation.

**Fig. 6:**
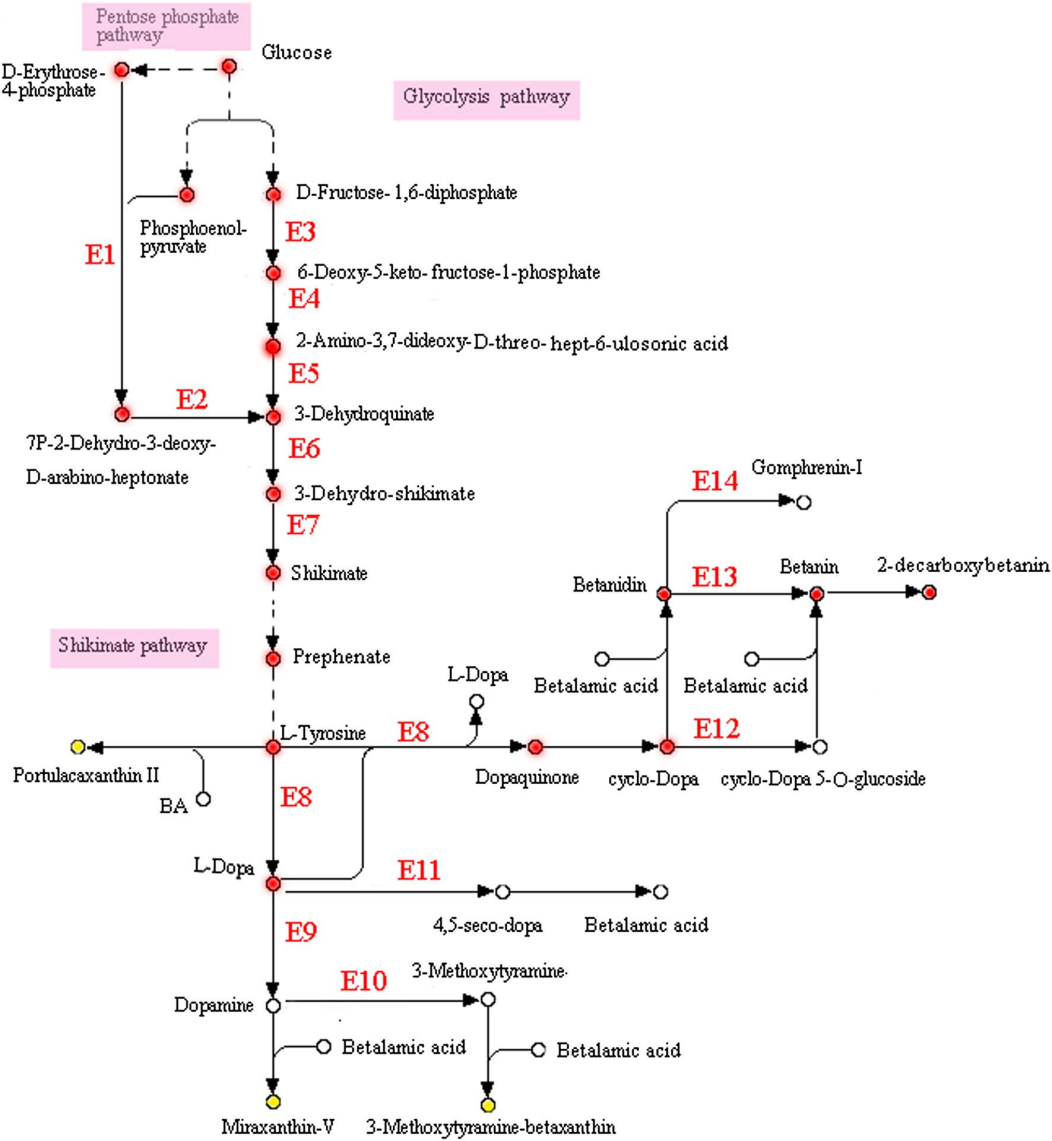
Simplified 2-decarboxybetanin biosynthetic pathway generated by KEGG enrichment analysis in *P. novae*-*zelandiae*. E1: 3-deoxy-7-phosphoheptulonate synthase [EC:2.5.1.54]; E2: 3-dehydroquinate synthase [EC:4.2.3.4]; E3: 6-deoxy-5-ketofructose-1-phosphate synthase [EC:2.2.1.11]; E4: 2-amino-3,7-dideoxy-D-threo-hept-6-ulosonate synthase [EC: 2.2.1.10]; E5: 3-dehydroquinate synthase II [EC:1.4.1.24]; E6: 3-dehydroquinate dehydratase I [EC:4.2.1.10]; E7: shikimate dehydrogenase [EC:1.1.1.25]; E8: tyrosinase [EC:1.14.18.1]; E9: aromatic-L-amino-acid decarboxylase [EC:4.1.1.28]; E10: catechol O-methyltransferase [EC:2.1.1.6]; E11: 4, 5-DOPA dioxygenase extradiol [EC:1.13.11.-]; E12: cyclo-DOPA 5-O-glucosyltransferase [EC:2.4.1.-]; E13: betanidin 5-O-glucosyltransferase [EC:2.4.1.-]; E14: betanidin 6-O-glucosyltransferase [EC:2.4.1.-]

KEGG pathway analysis also revealed the synthetic pathway of betalains. *P. novae*-*zelandiae* possesses a complete tyrosine synthetic pathway (Additional file 1: Figure S1). Thus, the addition of tyrosine could improve the pigment yield given that tyrosine is an important precursor for betalain biosynthesis. Additional file 1: Figure S2 reveals that the genes coding the enzymes (EC: 1.14.18.1; EC: 4.1.1.28; and EC: 2.1.1.6) were significantly up-regulated, and these up-regulated genes led us to investigate why red pigments did not appear during shake cultivation. Figure 6 exhibits a simplified 2-decarboxybetanin biosynthetic pathway. Notably, betalains are unstable substances that can easily be converted into 2-decarboxybetanin by decarboxylate reaction under acidic, thermally treated, or other conditions (Herbach et al. 2006; Wybraniec et al. 2013). The synthetic pathway found in *P. novae*-*zelandiae* means that betalain production by fungal fermentation is theoretically feasible.

### Validation of the gene expression profile

**Fig. 7:**
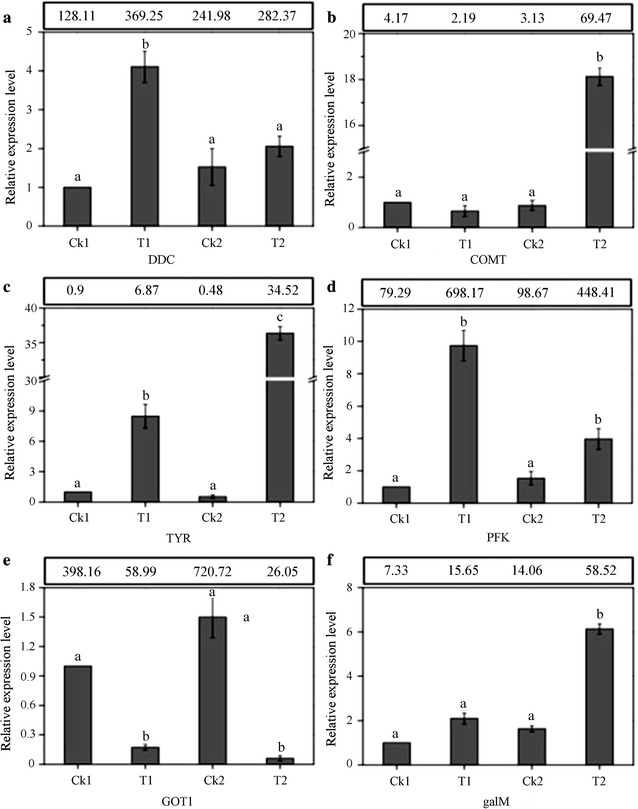
Validation of DEGs identified in RNA squencing analysis. **a** DDC; **b** COMT; **c** TYR; **d** PFK; **e** GOT1 and **f** galM. The RT-qPCR data represent the mean ± standard error of three biological replicates. Different lower case letter (a, b, and c) indicates the significant difference among Ck1, T1, Ck2 and T2 at P < 0.05. The reads per million reads (RPKM) determined using RNA squencing is shown in the block above each genes. Relative transcript levels are calculated by RT-qPCR with β-tubulin as the standard

To verify the quantitative results of the RNA sequencing experiments, six genes were selected for RT-qPCR analysis on the basis of their expression levels in sequencing data and importance in the regulation of betalain biosynthesis. Among them, five genes were significantly up-regulated, and one was significantly down-regulated. The results support the validity of transcriptomic sequencing (Fig. 7) and confirm that the expression profiles of genes, under the different cultivations determined by RT-qPCR, were similar to those of transcriptomic analysis.

In this work, the red-colored pigment, 2-decarboxybetanin, produced by the biomembrane surface fermentation of *P. novae*-*zelandiae*, was detected by high-resolution mass spectrometry, and transcriptomic analysis demonstrated the metabolic profile of *P. novae*-*zelandiae* in response to different cultivations and revealed the complete synthetic pathway of 2-decarboxybetanin in *P. novae*-*zelandiae*. These results suggest the possibility and feasibility of production of betalain by *P. novae*-*zelandiae* fermentation. This study is the first report about the production of betalains by microbial fermentation, and further work should focus on the improvement of 2-decarboxybetanin yield by optimization of fermentation factors or by metabolic regulation technology. In addition, the precise quantification of 2-decarboxybetanin should also be considered in the future considering that a small quantity of colorless compounds might exist in the 2-decarboxybetanin sample we purified.

## Additional file

**Additional file 1.** Additional figures and tables.
