# Supplementary material for: Feasibility and transcriptomic analysis of betalain production by biomembrane surface fermentation of Penicillium novae-zelandiae
Source: AMB Express. 2018 Jan 8;8:4. doi: 10.1186/s13568-017-0529-4 (PMC5758489; doi:10.1186/s13568-017-0529-4)
Supplement: Supplementary file 1 — Additional file 1. Additional figures and tables. [file 13568_2017_529_MOESM1_ESM.pdf]

SUPPLEMENTARY MATERIAL

Title: Feasibility and transcriptomic analysis of betalain production by biomembrane surface fermentation of *Penicillium novae-zelandiae*

Journal: AMB Express

Authors: Wang Hailei [1,2], Li Yi[1], Zhang Kun [1], Ma Yingqun[2], Li Ping[1, *]

Affiliations:

1: Henan Province Engineering Laboratory for Bioconversion Technology of Functional Microbes, College of Life Sciences, Henan Normal University, Xinxiang 453007, China;

2: Advanced Environmental Biotechnology Center, Nanyang Environment and Water Research Institute, Nanyang Technological University, Singapore 637141, Singapore;

*** Corresponding author:**

E-mail address: liping2062@163.com

Tel.: +86 3733326340; Fax: +86 3733326916.

Table S1 Mass spectrometric data of 2-Decarboxybetanin

2-Decarboxybetanin	Spectrometric data
Identified by	MS/MS .
Chemical formula	$C_{23}H_{27}N_2O_{11}$
λ_{max} (nm)	539
Retention time (s)	75.1
m/z of $[M+H]^+$	508.45
m/z from MS/MS of $[M+H]^+$	345.29

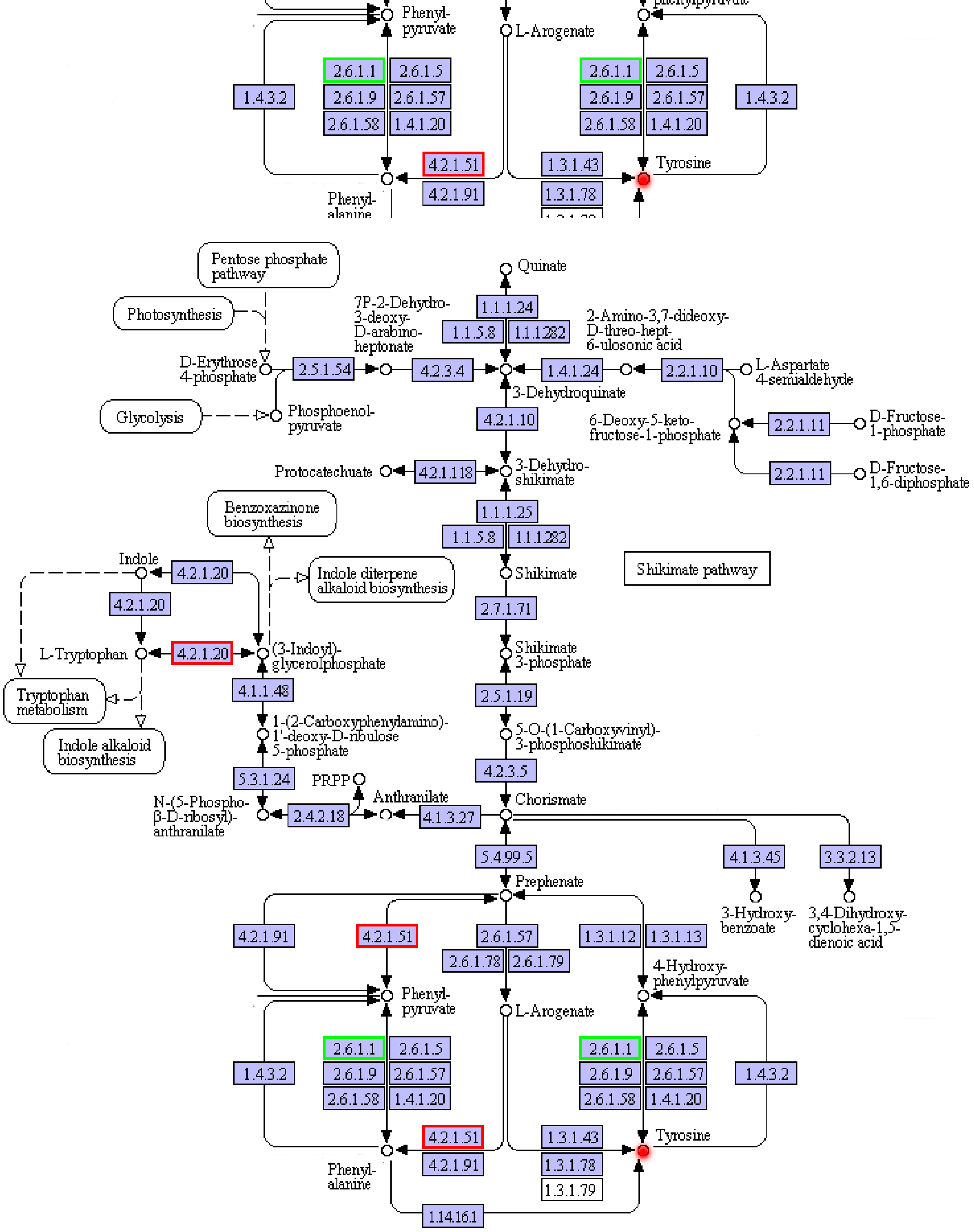

Fig.S1 Tyrosine biosynthesis pathway generated by KEGG enrichment analysis. The blue background of boxes indicates the genes were detected; The up-regulated and down-regulated genes were shown in red and green boxes, respectively.

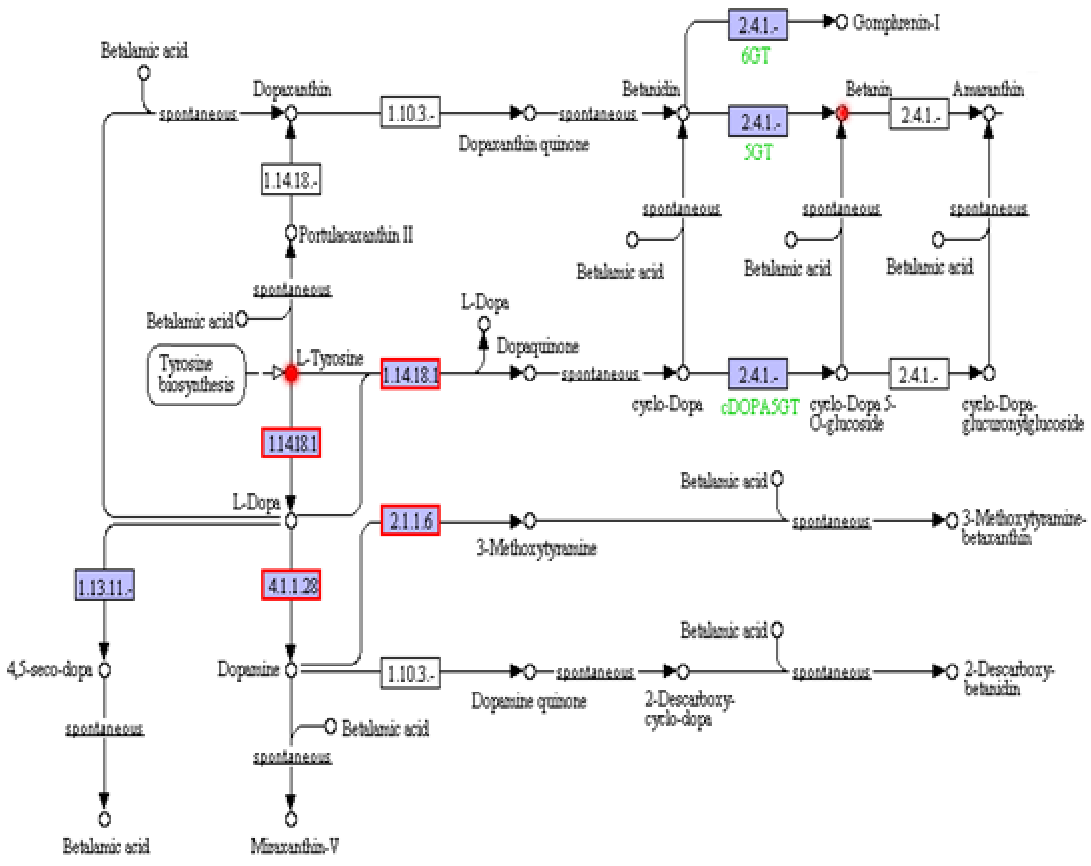

Fig.S2 Betanin biosynthesis pathway from tyrosine generated by KEGG enrichment analysis.

The blue background of boxes indicates the genes were detected;

The up-regulated and down-regulated genes were shown in red and green boxes, respectively.
